# Bovine Herpesvirus-1 Glycoprotein M Mediates the Translocation to the Golgi Apparatus and Packaging of VP8

**DOI:** 10.3390/v14091985

**Published:** 2022-09-08

**Authors:** Soumya Sucharita, Suresh Tikoo, Sylvia van Drunen Littel-van den Hurk

**Affiliations:** 1 Biochemistry, Microbiology and Immunology, University of Saskatchewan, Saskatoon, SK S7N 5E5, Canada; 2 Vaccine and Infectious Disease Organization, University of Saskatchewan, Saskatoon, SK S7N 5E3, Canada; 3 School of Public Health, University of Saskatchewan, Saskatoon, SK S7N 2Z4, Canada

**Keywords:** bovine herpesvirus-1, tegument, VP8, glycoprotein M, Golgi localization

## Abstract

VP8, the most abundant tegument protein of bovine herpesvirus-1 (BoHV-1), plays an important role in viral replication. According to our previous studies, VP8 localizes to the Golgi apparatus of BoHV-1-infected cells where it can be packaged into the virus; however, Golgi localization of VP8 does not occur outside of the context of infection. The goal of this study was to identify the viral factor(s) involved in the tropism of VP8 towards the Golgi. VP8 was found to interact with glycoprotein M (gM), and the VP8 and gM domains that are essential for this interaction were identified. VP8 and gM colocalized to the Golgi apparatus in BoHV-1-infected cells. In cells co-transfected with VP8- and gM-encoding plasmids, VP8 was also found to be localized to the Golgi, suggesting gM to be sufficient. The localization of VP8 to the Golgi was lost in cells infected with a gM deletion mutant, and the amount of VP8 incorporated into mature virus was significantly reduced. However, with the restoration of gM in a revertant virus, the localization to the Golgi and the amount of VP8 incorporated in the virions were restored. These results indicate that gM plays a critical role in VP8 subcellular localization to the Golgi and packaging into mature virions.

## 1. Introduction

Bovine herpesvirus-1 (BoHV-1) of the subfamily *Alphaherpesvirinae* is an important pathogen in cattle. It causes infectious bovine rhinotracheitis, conjunctivitis, balanoposthitis, and vulvovaginitis [1], which may lead to infertility, abortion, and reduced milk production [2]. Structurally, BoHV-1 is composed of an icosahedral proteinaceous capsid with a diameter of 125 nm. The 135 kb double-stranded DNA genome is packaged inside the capsid [3]. Approximately 20 proteins create a layer known as the tegument around the capsid, which is characteristic of herpesviruses. The viral envelope is made up of lipids and glycoproteins [4]. The tegument is an essential layer because tegument proteins can perform numerous vital roles in the life cycle of herpesviruses, including replication, virus assembly, and immune evasion. However, the tegument of many of the herpesviruses remains poorly characterized [5].

VP8, encoded by the *U_L_47* gene, is the most abundant tegument protein in BoHV-1 and is critical for virus replication and involved in induction of immune responses in the host [4,6]. It also plays a role in the alteration of the host defense responses and apoptosis of host cells [7]. A recombinant BoHV-1 that does not express VP8 does not replicate in cattle; in vitro replication of this deletion mutant is also impaired leading to a 100-fold reduction of intracellular virus and a 1000-fold reduction of extracellular virus in cell culture [4]. Previously, we reported that phosphorylation of VP8 by viral as well as host kinases plays an indispensable role in the functions and subcellular localization of VP8 [8]. Due to the presence of nuclear localization signals (NLS) [9], VP8 enters into the nucleus at the early stages of infection. Inside the nucleus, VP8 causes recruitment and redistribution of promyelocytic leukemia protein, which in turn inhibits host antiviral responses [10]. In transfected cells, the nuclear VP8 causes an accumulation of lipid droplets and increases cellular cholesterol [11]. VP8 was found to move to the cytoplasm at later stages of infection after phosphorylation by viral kinase US3 and to translocate to the Golgi, where the final packaging into the mature virus occurs. In cells co-transfected with VP8 and US3, VP8 also moves from the nucleus to the cytoplasm but is not transported to the Golgi in the absence of other viral factors [11].

The aim of this study was to identify the viral factor(s) responsible for the subcellular localization of VP8 to the Golgi. Glycoprotein M (gM) was found to interact with VP8, and the domains of gM and VP8 that are involved and essential for the interaction of these two proteins were identified. The translocation of VP8 to the Golgi was found to be mediated by gM, both within and outside the context of infection. In cells infected with a gM-deleted virus (ΔgM BoHV-1), VP8 remained cytoplasmic, and the amount of VP8 incorporated into mature virus was significantly reduced. This further supports a critical role of gM in the translocation of VP8 to the Golgi apparatus and packaging into the virions.

## 2. Materials and Methods

### 2.1. Cells and Viruses

Madin–Darby bovine kidney (MDBK) cells, embryonic bovine tracheal (EBTr) cells, and fetal bovine testicular (FBT) cells were grown in Eagle’s minimum essential medium (MEM; Sigma-Aldrich Canada Ltd., Oakville, ON, Canada), supplemented with 10 mM N-2-hydroxyethylpiperazine-N-2-ethane sulfonic acid (HEPES, Gibco, Life Technologies, Burlington, ON, Canada), 1% nonessential amino acids (Gibco, Life Technologies), 50 µg/mL gentamycin (Gibco, Life Technologies), and 10% fetal bovine serum (FBS; Life Technologies). The cells were grown in a 37 °C incubator supplied with 5% CO_2_.

Wild-type (WT) BoHV-1, ΔgM-BoHV-1 (BoHV-1 devoid of the *U_L_10* gene), and gM revertant BoHV-1 (ΔgM Rev BoHV-1) were propagated in MDBK cells. The titers were determined in MDBK cells in 24-well plates after overlay with 0.8% UltraPure low-melting agarose (Invitrogen/Thermo Fisher Scientific, Waltham, MA, USA) in MEM, and the virus stocks were stored at −80 °C.

Cell monolayers at 85–90% confluency were infected with WT BoHV-1, ΔgM-BoHV-1, or ΔgM Rev BoHV-1 at different multiplicities of infection (MOIs) in MEM. Virus was removed and replaced with MEM containing 2% FBS after 1.5 h. Cells and culture media were collected at different time points and centrifuged at 3000× *g* for 30 min at 4 °C.

### 2.2. Plasmids

pFLAG-VP8, pFLAG-ΔNLS VP8, and plasmids encoding truncated versions of VP8 with FLAG tag were used as described previously [12]. pHA-gM was constructed by amplification of the *U_L_10* gene from the BoHV-1 genome using 3′ATTGAATTCATGGCGGGCTCCGCGCAGCCTG 5′ and 3′ATTTCTAGATTTGACGTGCGCGGGGGGTGGG 5′ primers, and ligation into *Xba*I- and *EcoR*I-digested pcDNA3.1HA (Addgene, Water-town, MA, USA). 

pHA-gM deletion mutants were constructed similarly by amplifying desired segments of the *U_L_10* gene from the BoHV-1 genome using primers shown in Table 1. The amplified segments were ligated into pcDNA3.1HA (Addgene) digested with *Xba*I and *EcoR*I.

### 2.3. Antibodies

Antibodies, including mouse monoclonal VP8-specific antibody (clone 1G4 2G2) [6] and rabbit polyclonal VP5-specific antibody [4], were used as described previously. Antibodies specific for glycoproteins B, C, D [13], and H [14] were used as described. 

Antibodies specific for glycoproteins E, N, and L were produced in-house by using synthetic peptides according to the protocol described by Labiuk et al. [12]. To produce rabbit gM-specific antibodies, a blunt end repaired (*MSc*I-*PpuM*I) 265 bp DNA fragment of the *U_L_10* Orf (containing C-terminal 80 amino acids) was ligated to *Xma*I-digested (blunt-end repaired) plasmid pGEX-2T DNA creating plasmid pGEXUL10. GST-UL10 fusion protein was produced by induction of plasmid pGEXUL10 transformed *E.coli* with 0.1 mM isopropyl -b-D-thiogalactoside (IPTG) and purified by sodium dodecyl sulphate (SDS)-polyacrylamide gel electrophoresis (PAGE). Rabbits were immunized subcutaneously with 300 µg of fusion protein in Freund’s complete adjuvant. The rabbits were boosted with 100 µg of fusion protein in Freund’s incomplete adjuvant at 4, 8, and 12 weeks after primary injection. The rabbits were bled 10 days after the last boost and sera were collected. 

HA tag-specific rabbit polyclonal antibody (Cell Signaling Technology, Danvers, MA, USA) and FLAG tag-specific mouse monoclonal antibody (Sigma-Aldrich Canada Ltd.) were used to detect HA-tagged and FLAG-tagged proteins, respectively. Mouse monoclonal antibody Golgi 58K and rabbit polyclonal antibody GolgB1 specific for Cis-Golgi (Sigma-Aldrich) were used to detect the Golgi apparatus. For Western blotting, IRDye 680RD goat anti-rabbit IgG, IRDye 680 RD goat anti-mouse IgG, IRDye 800RD goat anti-rabbit IgG, and IRDye 800RD goat anti-mouse IgG (Li-Cor Biosciences, Lincoln, NE, USA) were used. Alexa 488-conjugated goat anti-mouse IgG and Alexa 633-conjugated goat anti-rabbit IgG (Invitrogen/Thermo Fisher Scientific) were used for immunofluorescent staining.

### 2.4. Transfection

Monolayers of EBTr cells at 80–90% confluency were transfected with a total amount of 3 µg plasmid(s) (pFLAG-VP8, pHA-gM, pFLAG-VP8 deletion mutants, pHA-gM deletion mutants, pFLAG-ΔNLS VP8) using Lipofectamine 3000 transfection reagent (Invitrogen/Thermo Fisher Scientific), and cells were collected at 24–48 h post transfection.

### 2.5. Preparation of Cell Lysates

Infected MDBK cells and transfected EBTr cells were collected using ice-cold phosphate-buffered saline, pH 7.4 (PBS) (Gibco, Life technologies), at different time intervals and centrifuged at 8000 × *g* for 10 min. Cell pellets were lysed for 40 min on ice in RIPA buffer (50 mM Tris-HCl, 150 mM NaCl, 1 mM EDTA, 1% Triton-X100, pH 7.4) and protease inhibitor cocktail (Sigma-Aldrich Canada Ltd.) in a 10:1 *v*/*v* ratio. Cell lysates were clarified by centrifugation at 12,000× *g* for 10 min at 4 °C. The supernatants were collected and stored at −20 °C for further use.

### 2.6. Immunoprecipitation and Western Blotting

Infected cell lysates (prepared as described above) were incubated with VP8-specific or gM-specific antibodies overnight at 4 °C, followed by Protein G Sepharose Fast Flow beads (GE HealthCare, Niskayuna, NY, USA) for 5 h at 4 °C. Transfected cell lysates were incubated with Anti-FLAG M2 Affinity Gel (Sigma-Aldrich Canada Ltd.) or anti-HA agarose (Pierce/Thermo Fisher Scientific) and incubated at 4 °C overnight. Lysates were washed 3 to 4 times with wash buffer (0.05 M Tris-HCl, 0.15 M NaCl, pH 8) and eluted with SDS loading dye at 95 °C for 2 min. SDS loading dye was also added to a fraction of the whole cell lysates, which were then treated at 95 °C for 2 min before being used as input control. These samples were subjected to SDS-polyacrylamide gel electrophoresis on 8% or 15% gels or 4–15% gradient gels and transferred to 0.45 µm nylon membranes (Bio-Rad, Hercules, CA, USA) at 100 Volt for 1 h. This was followed by blocking the membrane with 3% blotto (3 g milk powder in TBST (20 mM Tris-HCl, 150 mM NaCl, 0.1% *w*/*v* Tween 20, pH 7.4)) for 1 h and incubation with respective antibodies overnight. The following day, membranes were washed with TBST and incubated with IRDye 680RD goat anti-mouse IgG or IRDye 800RD goat anti-rabbit IgG (LI-COR Biosciences, Lincoln, NB, USA) for 2–3 h. An Odyssey Infrared Imaging System and Odyssey 3.0.16 application software (LI-COR Biosciences) were used for detection and analysis. Quantification of the protein bands was performed by using Image Studio Lite version 5.2. Intensities of the protein bands were reported in comparison to the intensities of the protein bands of WT BoHV-1.

### 2.7. Construction of Recombinant Virus

**Figure 1:**
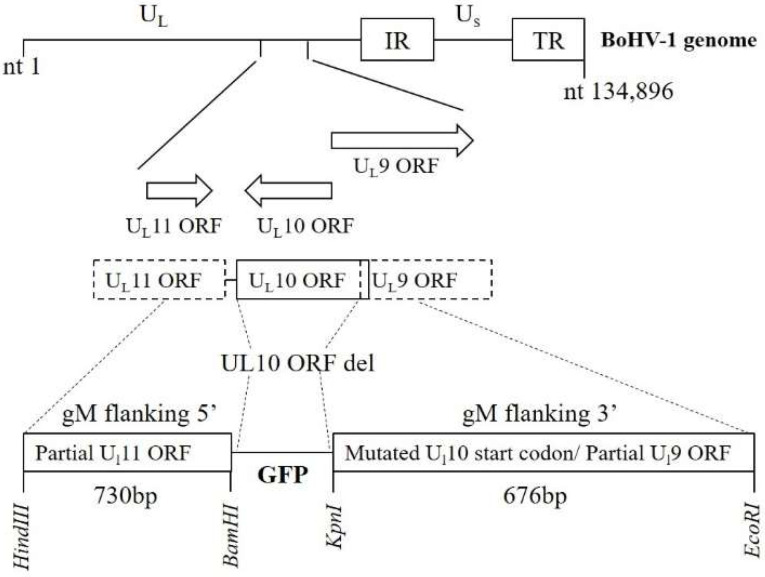
Schematic of the plasmid constructed for deletion of the *U_L_10* gene. On top, the BoHV-1 genome consisting of unique long (UL) and unique short (US) regions along with internal repeats (IR) and terminal repeats (TR) is depicted. The localization of *U_L_9, U_L_10* (gM), and *U_L_11* genes is shown. In the constructed plasmid, the gM gene is deleted and GFP is inserted in that position with the insertion of *BamH*I and *Kpn*I sites. The GFP was flanked with a 730bp 5′ gM flanking region and 676 bp 3′ gM flanking region along with a mutated start codon for *U_L_10*.

Plasmids were constructed by use of primers listed in Table 2. A pEGFP-N1 plasmid was cut with *Nhe*I and *Age*I and self-ligated using T4 polymerase to remove the multiple cloning site. The GFP cassette from the resulting plasmid was amplified by primer 1 and primer 2 and cloned into pUC57 cut with *BamH*I and *Kpn*I to construct pUC-GFP. Three prime regions of the *U_L_10* gene were amplified from the BoHV-1 genome and cloned into pUC-GFP, which was then digested with *Kpn*I and *EcoR*I to construct pGFP-UL10. To mutate the *U_L_10* start codon, two segments were amplified using pairs of primers 5, 6 and primers 7, 8. These segments were later joined together using primers 5 and 7. The joined segment was cloned into the pUC-UL10 vector digested with *Hind*III and *BamH*I, which resulted in pGFP-UL10del. Primers 3 and 5 were used to amplify the modified *U_L_10* region from pGFP-UL10del, and the resultant PCR fragment was transfected with the WT BoHV-1 genome for homologous recombination in FBT cells using Lipofectamine 3000 (Invitrogen). Viral plaques expressing GFP were plaque-purified and ΔgM BoHV-1 stocks were prepared. A schematic for the construction of ΔgM BoHV-1 is shown in Figure 1.

For construction of ΔgM Rev BoHV-1, the *U_L_10* gene with the flanking homologous regions was amplified from the WT BoHV-1 genome using primers 3 and 5, and the amplified product was transfected with the ΔgM BoHV-1 genome for homologous recombination in FBT cells. Plaques that did not express GFP were plaque purified to prepare ΔgM Rev BoHV-1 stocks.

### 2.8. Viral Growth Kinetics

MDBK cells were seeded in 35 mm dishes and infected at 80–90% confluency with WT BoHV-1, ΔgM BoHV-1, or ΔgM Rev BoHV-1 at a MOI of 0.1. Cells and culture media were collected at 0, 5, 10, 15, 20, 25, 30, and 35 h post infection. Cells were put through 2 freeze–thaw cycles to release the virus. The titers of the virus released from the cells (intracellular) and the virus from the culture media (extracellular) were determined in MDBK cells in a 24-well plate at different time points.

### 2.9. Confocal Microscopy

Cells were seeded in 2-well Permanox Lab-Tek chamber slides (Nunc, Thermo Fisher) and infected at 80–90% confluency with WT BoHV-1, ΔgM BoHV-1, or ΔgM Rev BoHV-1 at various MOIs. Cells were fixed at different times post infection with 4% paraformaldehyde (Sigma-Aldrich Canada Ltd.), washed with phosphate buffered saline (10 mM Na_2_HPO_4_, 1.8 mM KH_2_PO_4_, 137 mM NaCl, 2.7 mM KCl, pH 7.4 (PBS)), and then blocked overnight with 1% goat serum. The cells were permeabilized the following day with 0.1% Triton X-100. Subsequently, they were incubated for 2–3 h at room temperature (22 ± 1 °C) with respective primary antibodies prepared in 1% goat serum, followed by Alexa 488-conjugated goat anti-rabbit or goat anti-mouse IgG, Alexa 633-conjugated goat anti-rabbit or goat anti-mouse IgG, or both Alexa 488- and Alexa 633-conjugated IgG (Invitrogen, Thermo Fisher), for 1–2 h at room temperature. Finally, the cells were treated with Prolong Gold with DAPI (Invitrogen, Thermo Fisher). A Leica SP8 confocal microscope (Leica Microsystems, Wetzlar, Germany) was used to analyze the cells.

### 2.10. Transmission Electron Microscopy (TEM)

Monolayers of 80–90% confluent MDBK cells were infected with WT BoHV-1, ΔgM BoHV-1, or ΔgM Rev BoHV-1 at a MOI of 5. When all the cells showed a rounded morphology, the monolayers were washed with PBS (Gibco, Life Technologies) and fixed with 2% glutaraldehyde (Electron microscopy Sciences, Hatfield, PA, USA) and 0.1M sodium cacodylate (NaCac) (Canemco, Gore, QC, Canada), pH 7.3, for 3–5 h at 4 °C. The fixative along with the cells were collected and the remaining cells were gently scraped off, and the suspension was centrifuged at 300 × *g* for 5 min at 4 °C. The supernatant was discarded, and the pellet was resuspended in 0.1 M NaCac. The fixed cells were then centrifuged, and the pellet was mixed with warm 1% low-melting agarose. The cells were centrifuged again and placed in the refrigerator to allow the agarose to solidify. Subsequently, the agarose was removed from the centrifuge tubes and the excess agarose was removed from the cell pellets. The pellets were then placed in 0.1M NaCac at 4° overnight. Samples were fixed in freshly prepared osmium tetraoxide (Fisher scientific) (1% OsO4 in 0.1M NaCac), for 1 h at room temperature. Samples were rinsed with water, dehydrated, and stained with ethanol and uranyl acetate (UrAc) (Greenfield Global, Brampton, ON, Canada) at room temperature as follows: 50% ethanol, 5–10 min; UrAc in 70% ethanol, 1 h; 70% ethanol, 5–10 min; 95% ethanol, 5–10 min; and three times with 100% ethanol, 5–10 min. This was followed by three rinses in propylene oxide. Subsequently, infiltration in Epon/Araldite was performed as follows: 1 part Epon/Araldite (Electron Microscopy Sciences); 2 parts propylene oxide (Thermo fisher) for 30 min: 2 parts Epon/Araldite: 1 part propylene oxide for 1–2 h; and pure Epon/Araldite overnight. Samples were then oriented in molds and fresh Epon/Araldite was added. The samples were polymerized at 60 °C for 24 h. Blocks were sectioned to around 90 nm with a diamond knife and were placed on copper grids for observation under the Hitachi HT7700 (Tokyo, Japan) transmission electron microscope.

## 3. Results

### 3.1. Glycoprotein M Interacts with VP8

**Figure 2:**
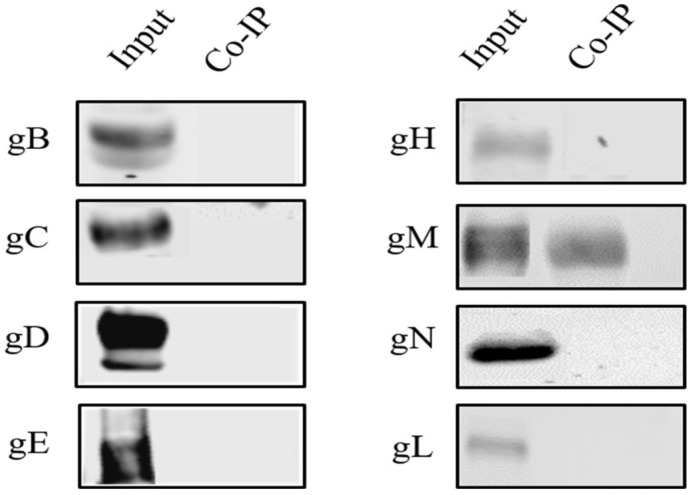
Identification of the glycoprotein interacting with VP8. Lysates from MDBK cells infected with WT BoHV-1 were incubated with rabbit VP8-specific antibody followed by Protein Sepharose G beads. After elution by SDS loading dye, the samples were subjected to SDS-polyacrylamide gel electrophoresis on an 8% (gB, gC, gD, gE, gH, gM) or 15% (gN, gL) gel and transferred to 0.45 µm nylon membranes. Glycoproteins were detected with antibodies specific for gB, gC, gD, gE, gH, gM, gN, or gL, followed by IRDye 680RD goat anti-mouse IgG and IRDye 800RD goat anti-rabbit IgG. Glycoprotein M was found to precipitate with VP8. A fraction of the whole cell lysate was used as input control. Glycoproteins B, gC, gD, gE, gH, gM, gN, and gL were all present in the infected cell lysate.

Previously, we showed that VP8 localizes to the Golgi during viral infection but not after transfection, suggesting that a viral component is involved in its translocation to the Golgi. To identify the component(s) responsible for the translocation of VP8 from the cytoplasm to the Golgi, a potential interaction of VP8 with one of the BoHV-1 glycoproteins was investigated. MDBK cells were infected with WT BoHV-1 at a MOI of 5, and the cells were collected at 18 h post infection. Cell lysates were incubated with VP8-specific monoclonal antibodies overnight at 4 °C, followed by Protein G Sepharose beads and elution with SDS loading dye. The cell lysates were analyzed by Western blotting and probing with antibodies specific to glycoproteins B, C, D, E, M, N, and L. Only glycoprotein M was found to co-precipitate with VP8 (Figure 2), implying that gM is the most probable candidate aiding in the translocation of VP8 from the cytoplasm to the Golgi.

**Figure 3:**
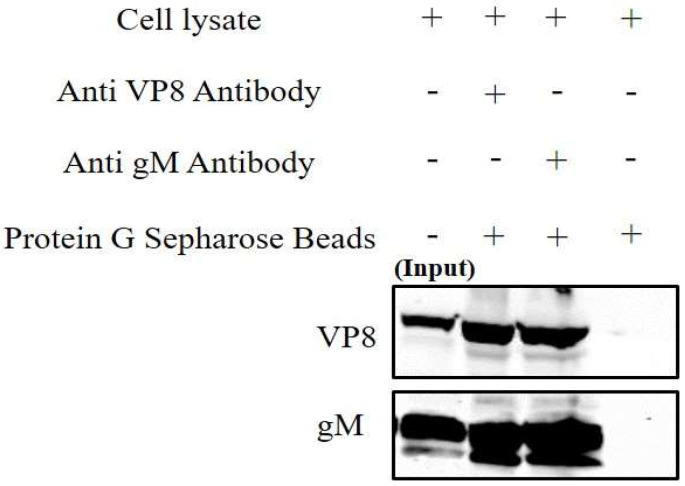
VP8 and gM interact in WT BoHV-1-infected cells. Cell lysates from MDBK cells infected with WT BoHV-1 were incubated with VP8-specific or gM-specific antibodies, followed by Protein Sepharose G beads or with Protein G Sepharose without antibodies. After elution by SDS loading dye, the samples were subjected to SDS-polyacrylamide gel electrophoresis on an 8% gel and transferred to 0.45 µm nylon membranes. A fraction of the whole cell lysates was used as input control. VP8 and gM were detected with monoclonal anti-VP8 and rabbit anti-gM antibodies, followed by IRDye 680RD goat anti-mouse IgG and IRDye 800RD goat anti-rabbit IgG, respectively.

To confirm the interaction between VP8 and gM, MDBK cells were infected with WT BoHV-1 at a MOI of 5, and the cells were collected at 18 h post infection. Subsequently, the cells were lysed and incubated with antibodies specific for either gM or VP8, followed by binding to Protein G Sepharose beads and elution in SDS loading dye at 95 °C. The eluates were analyzed by Western blotting, and antibodies specific for VP8 and gM were used for detection. This demonstrated that gM precipitates together with VP8 and vice versa (Figure 3), further supporting that there is an interaction between VP8 and gM. After incubation of the lysates with only Protein G Sepharose beads without antibodies, no gM or VP8 was found to be present.

**Figure 4:**
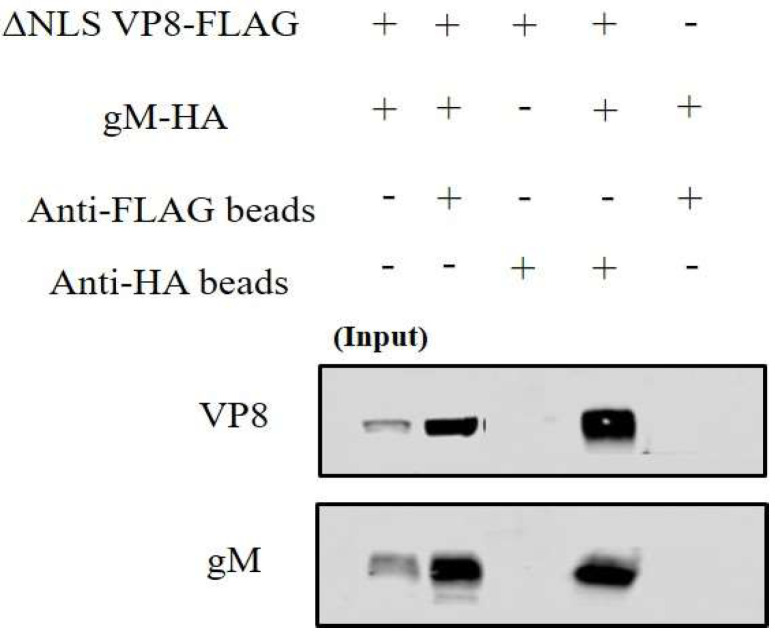
VP8-FLAG and gM-HA interact with each other in co-transfected cells. EBTr cells were co-transfected with ΔNLS VP8-FLAG and pgM-HA, or with ΔNLS VP8-FLAG or pgM-HA, and the lysates were incubated with anti-FLAG or anti-HA beads. After elution by SDS loading dye, the samples were subjected to SDS-polyacrylamide gel electrophoresis on an 8% gel and transferred to 0.45 µm nylon membranes. A fraction of the whole cell lysates was used as input control. VP8-FLAG and gM-HA were detected with monoclonal anti-FLAG and anti-HA antibodies, respectively, followed by IRDye 680RD goat anti-mouse IgG.

To confirm that the interaction between VP8 and gM is independent of other viral factors, ΔNLS VP8-FLAG and gM-HA plasmids were co-transfected into EBTr cells and incubated either with anti-FLAG or anti-HA resins before being eluted in SDS loading dye at 95 °C. Because VP8 moves into the nucleus in transfected cells and cannot move to the cytoplasm in the absence of US3, ΔNLS VP8-FLAG was used, which expresses VP8 without NLS, such that it stays cytoplasmic. The eluates were then subjected to Western blotting; antibodies specific for FLAG and HA tags were used to detect the ΔNLS VP8-FLAG and gM-HA proteins, respectively. As shown in Figure 4, gM-HA protein was detected in the eluate after incubation of the co-transfection lysate with anti-FLAG resin, while VP8-FLAG protein was detected in the eluate after incubation of the co-transfection lysate with anti-HA resin. No VP8 was found after incubation with anti-HA beads in absence of gM-HA, and no gM was found after incubation with anti-FLAG beads in absence of VP8-FLAG. This experiment confirms that VP8 and gM interact with each other and that this interaction is independent of other viral factors.

### 3.2. Glycoprotein M and VP8 Colocalize at the Golgi Apparatus

**Figure 5:**
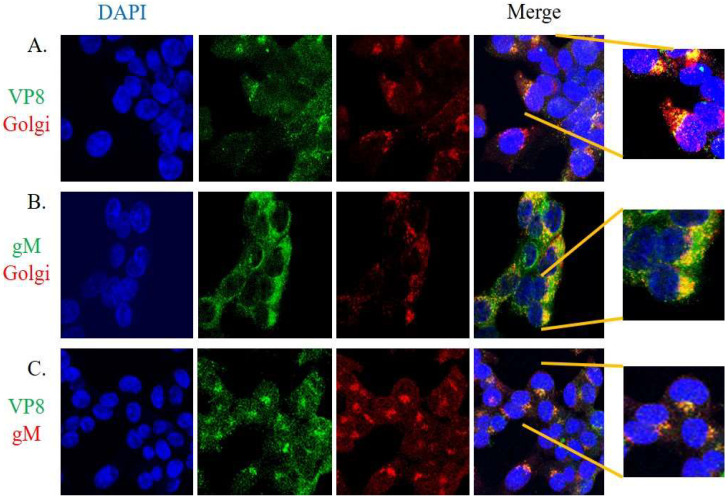
Co-localization of VP8 and gM at the Golgi in cells infected with WT BoHV-1. MDBK cells infected with WT BoHV-1 were fixed and incubated with antibodies specific for VP8, gM, or Golgi. Alexa 488-conjugated goat anti-rabbit or goat anti-mouse IgG and Alexa 633-conjugated goat anti-rabbit or goat anti-mouse IgG were used to detect the primary antibodies. (**A**) VP8 (green) and Golgi (red), and localization of VP8 in the Golgi in the merged image (yellow). (**B**) Glycoprotein M (green) and Golgi (red), and localization of gM in the Golgi in the merged image (yellow). (**C**) VP8 (green) and gM (red), and co-localization of VP8 and gM as shown in the merged image (yellow). The nuclei were stained with Prolong gold DAPI. Cells in the boxes are magnified 5X. The cells were examined with a Leica SP8 confocal microscope.

In order for gM to be the glycoprotein responsible for translocation of VP8 to the Golgi, VP8 and gM must colocalize at the Golgi. To investigate this, MDBK cells were infected with WT BoHV-1 at a MOI of 5, fixed with paraformaldehyde at 7 h post infection, and analyzed by confocal microscopy. Panel A of Figure 5 displays VP8 and Golgi, and the merged image shows the localization of VP8 in the Golgi at 7 h post infection. Panel 5B depicts the localization of gM in the Golgi at 7 h post infection. The merged image in Panel 5C indicates VP8 colocalizing with gM at 7 h post infection. From this three-way study, it can be confirmed that VP8 and gM colocalize in the Golgi at 7 h post infection, which suggests that gM supports the translocation of VP8 to the Golgi after its phosphorylation by US3 and export from the nucleus to the cytoplasm.

**Figure 6:**
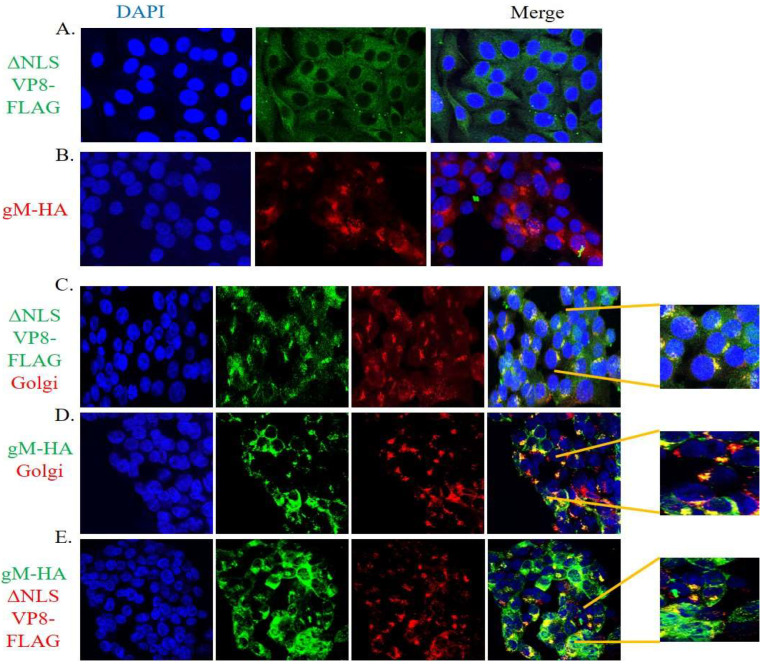
Localization of ΔNLS VP8-FLAG and gM-HA proteins in cells transfected with pΔNLS VP8-FLAG and pgM-HA individually (**A**,**B**) or in combination (**C**–**E**). EBTr cells transfected with ΔNLS VP8-FLAG and/or gM-HA were fixed and incubated with antibodies specific for VP8, gM, or Golgi. Alexa 488-conjugated goat anti-rabbit or goat anti-mouse IgG and Alexa 633-conjugated goat anti-rabbit or goat anti-mouse IgG were used to detect the primary antibodies. (**A**) Distribution of ΔNLS VP8-FLAG (green) in the cytoplasm. (**B**) Localization of gM-HA (red) in the Golgi. (**C**) ΔNLS VP8-FLAG (green), Golgi (red), and localization of VP8 in the Golgi in the merged image (yellow). (**D**) gM-HA (green), Golgi (red), and localization of gM in the Golgi in the merged image (yellow). (**E**) gM-HA (green) and ΔNLS VP8-FLAG (red), and co-localization of VP8 and gM as shown in the merged image (yellow). The nuclei were stained with Prolong gold DAPI. Cells in the boxes are magnified 5X. The cells were examined with a Leica SP8 confocal microscope.

To confirm the involvement of gM in the translocation of VP8 to the Golgi, three sets of EBTr cells were transfected with ΔNLS VP8-FLAG or gM-HA plasmids individually, or co-transfected with gM-HA and ΔNLS VP8-FLAG plasmids. Transfected cells were fixed at 24 h post transfection. Antibodies specific for VP8, gM, and Golgi were used to identify ΔNLS VP8-FLAG, gM-HA, and the Golgi apparatus, respectively. In Figure 6, panel A shows the distribution of VP8 in cells transfected with ΔNLS VP8-FLAG plasmid; in the absence of gM, localization of VP8 was predominantly cytoplasmic. Panel 6B shows the localization of gM in the Golgi in cells transfected with gM-HA plasmid. In the cells co-transfected with ΔNLS VP8-FLAG and gM-HA plasmids, the merged images show localization of ΔNLS VP8-FLAG (Panel 6C) and gM-HA (Panel 6D) in the Golgi. In panel 6E, co-localization of ΔNLS VP8-FLAG and gM-HA is shown. These results confirm that the presence of gM-HA is sufficient to mediate the translocation of VP8 to the Golgi.

### 3.3. A Domain between Amino Acids 538–632 in VP8 Interacts with a Domain between Amino Acids 210–300 in gM

**Figure 7:**
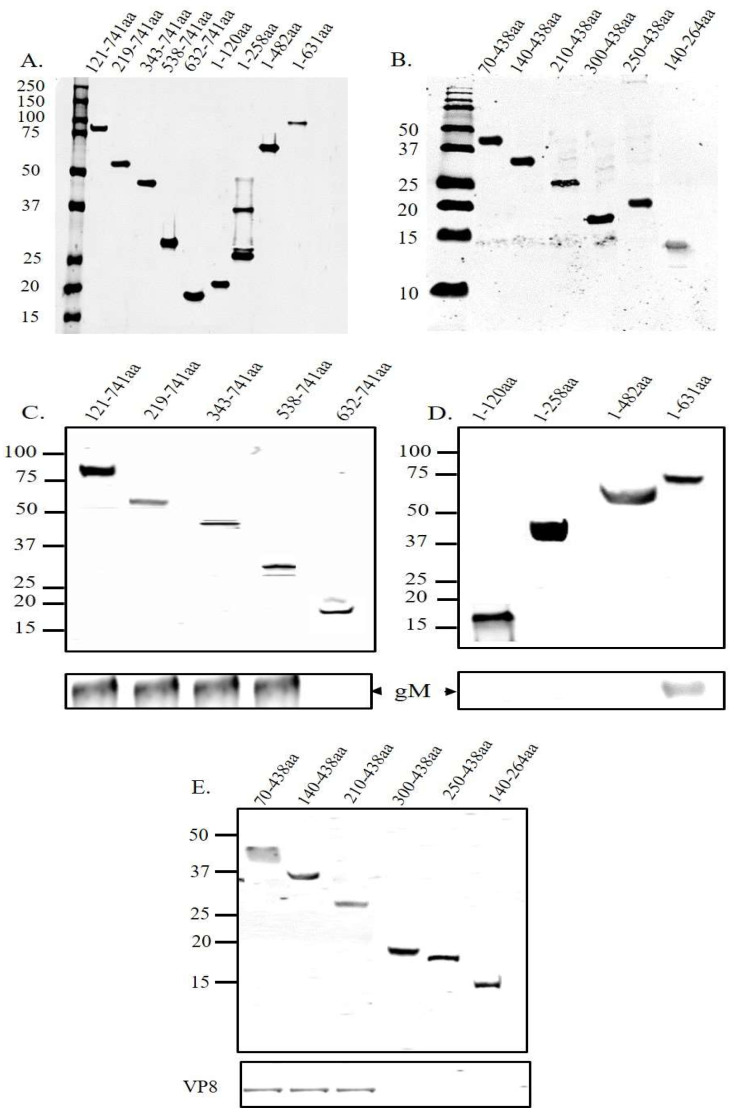
Identification of the interacting domains of VP8 and gM. EBTr cells were co-transfected with FLAG-tagged truncated VP8 versions and pgM-HA or with HA-tagged truncated gM versions and pFLAG-VP8, and the lysates were either directly analyzed by SDS-PAGE (**A**,**B**) or incubated with anti-FLAG (**C**,**D**) or anti-HA (**E**) beads. After elution by SDS loading dye, the samples were subjected to SDS-polyacrylamide gel electrophoresis on a 4–15% gel and transferred to 0.45 µm nylon membranes. (**A**) Expression of truncated versions of VP8 protein. (**B**) Expression of truncated versions of gM protein. (**C**) Immunoprecipitation of full-length gM-HA by N-terminal VP8 deletions shows an interaction of gM-HA with all VP8 versions except the deletion lacking amino acids 1–631. (**D**) Immunoprecipitation of full-length gM-HA by C-terminal VP8 deletions shows an interaction of gM-HA with VP8 consisting of amino acids 1-631, but not with the other VP8 versions. (**E**) Immunoprecipitation of full- length VP8-FLAG by truncated gM-HA proteins shows an interaction of VP8-FLAG in the presence of the amino acids 70–438, 140–438, and 210–438 of gM-HA. Molecular weight markers × 10^−3^ are shown in the left margins.

**Figure 8:**
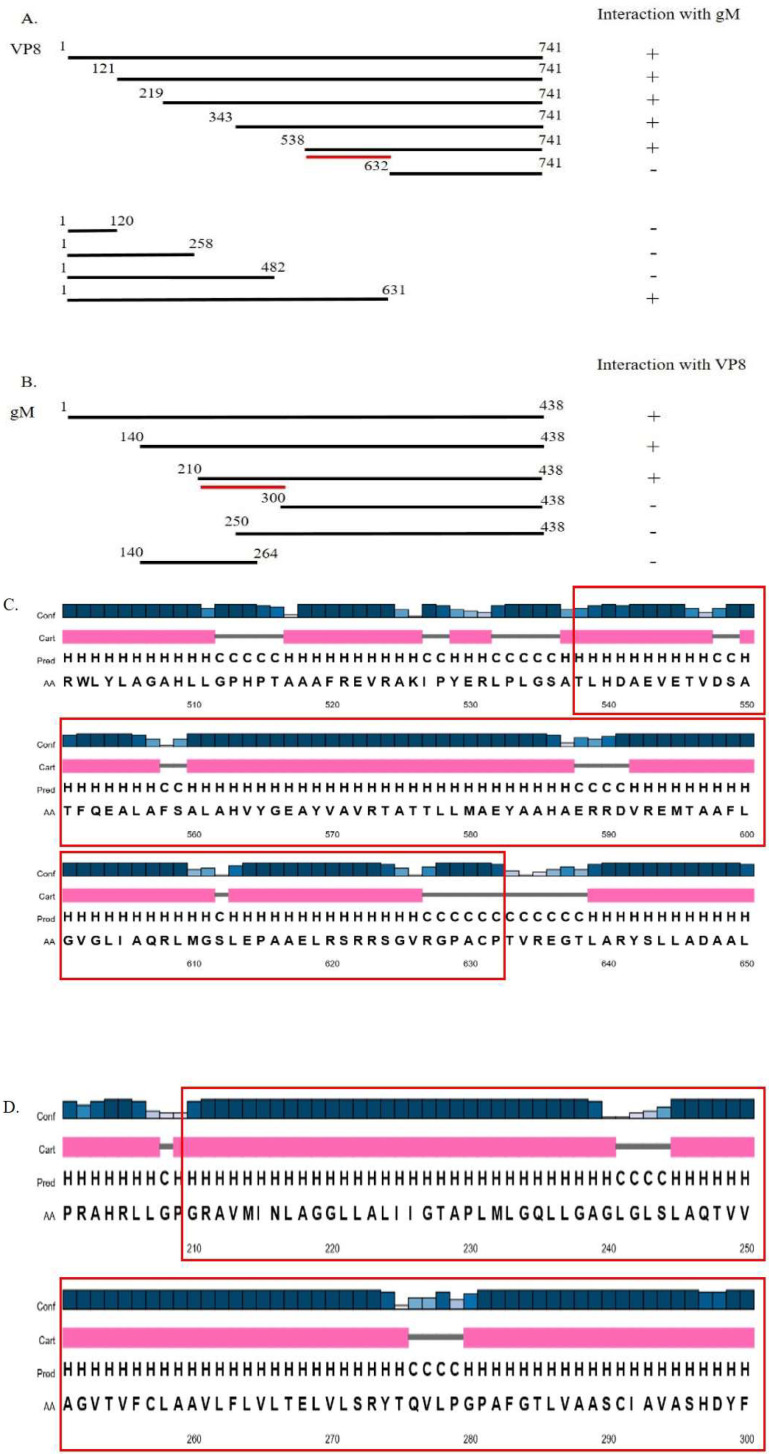
Representation of the domains of VP8 and gM that interact with each other. (**A**) Schematic representation of interaction between truncated versions of VP8-FLAG with full-length gM-HA. (**B**) Schematic representation of interaction between truncated versions of gM-HA with full-length VP8-FLAG. (**C**) Predicted secondary structure of the domain of VP8-FLAG (538–632) involved in the interaction with gM-HA. (**D**) Predicted secondary structure of the domain of gM-HA (210–300) involved in the interaction with VP8-FLAG.

To identify the interaction domains of VP8 and gM, several C-terminal and N-terminal deletions were made in the VP8 and gM proteins, and the truncated versions were cloned into a plasmid with FLAG and HA tag, respectively. EBTr cells were co-transfected with the FLAG-tagged VP8-deletion mutants and full-length pgM-HA or HA-tagged gM-deletion mutants and full-length pVP8-FLAG and collected at 24 h post transfection. The expression of the truncated VP8 and gM proteins is shown in Figure 7A,B, respectively. Lysates from cells cotransfected with VP8 deletion mutants and full-length gM-HA were incubated with anti-FLAG resin, and those from cells co-transfected with gM-HA deletion mutants and full-length VP8 FLAG were incubated with anti-HA resin. The lysates were then eluted in SDS loading dye at 95 °C. When the eluates were subjected to Western blotting, with HA- and FLAG-specific antibodies for detection, VP8 was found to interact with amino acids 210–300 of gM (Figure 7C,D), while gM interacted with amino acids 538–632 of VP8 (Figure 7E). Figure 8A,B show a schematic of the interaction between the respective truncated proteins and full-length proteins, and the interaction domains on VP8 and gM, respectively. Prediction of the secondary structure of VP8 and gM using PsiPred (http://bioinf.cs.ucl.ac.uk/psipred, accessed on 18 May 2022) shows that the region of amino acids identified to be responsible for the interaction between VP8 (538–632) (Figure 8C) and gM (210–300) (Figure 8D) contain helix–coil–helix structures, which are known to be characteristic of functional domains [15].

### 3.4. The ΔgM BoHV-1 Titer Is Significantly Reduced and Its Egress Is Affected

**Figure 9:**
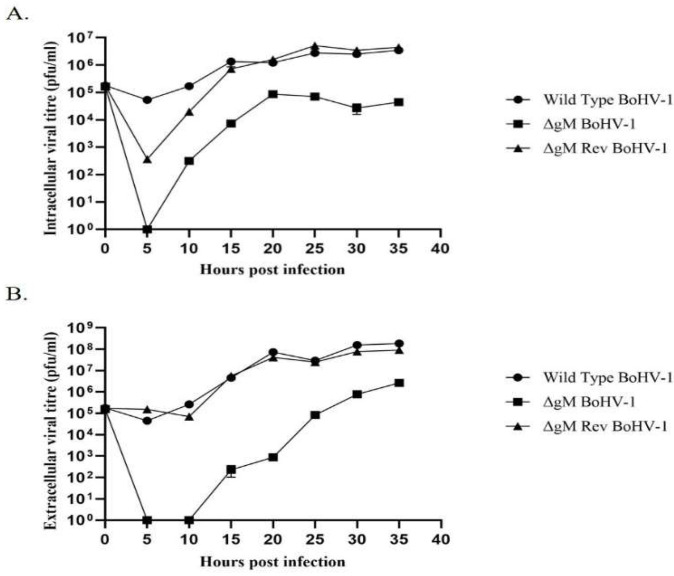
One-step growth curve of WT BoHV-1, ΔgM BoHV-1, and ΔgM Rev BoHV-1. MDBK cells were infected with WT BoHV-1, ΔgM BoHV-1, or ΔgM Rev BoHV-1 at a MOI of 0.1, and cells and supernatant media were collected at different times after infection. (**A**) Viral titer of the intracellular virus. (**B**) Viral titer of the extracellular virus. The error bars indicate standard deviation.

To test the requirement of gM for the translocation of VP8 to the Golgi, ΔgM BoHV-1 and ΔgM Rev BoHV-1 were constructed. To first compare its replication efficiency to WT BoHV-1, the ΔgM BoHV-1 recombinant virus was tested in a one-step growth curve. This showed that the growth rate of ΔgM BoHV-1 was slower than that of WT BoHV-1. The intracellular and extracellular virus in ΔgM BoHV-1-infected cells showed a ~100-fold reduction in the titer when compared to that of WT BoHV-1 (Figure 9). No infectious virus particles were detected inside the cells at 5 h post infection in ΔgM BoHV-1-infected cells (Figure 9A), and no virus particles were detected in the extracellular media up to 10 h post infection (Figure 9B), indicating a delay in viral assembly and particle egress from the cell.

### 3.5. ΔgM BoHV-1 Shows Delayed Egress Compared to WT BoHV-1 and ΔgM Rev BoHV-1

**Figure 10:**
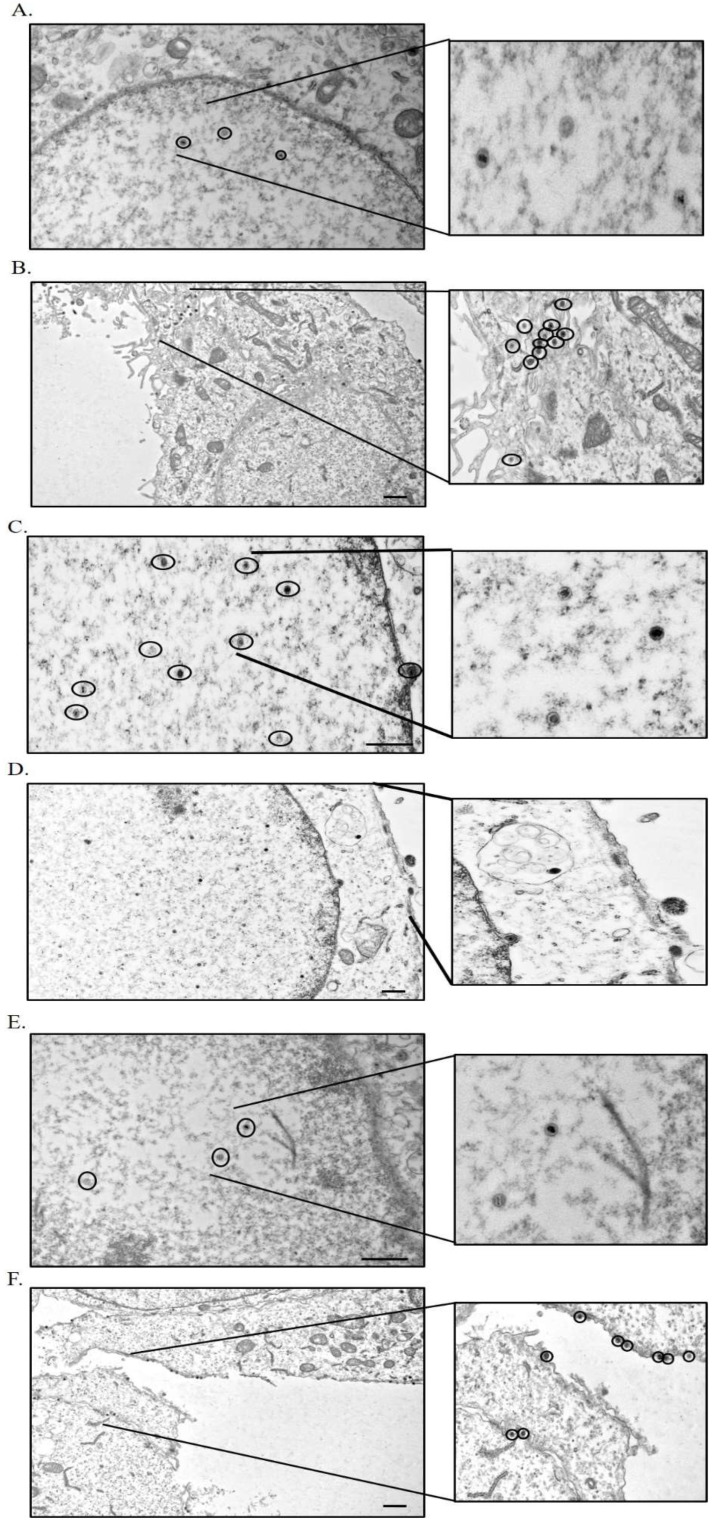

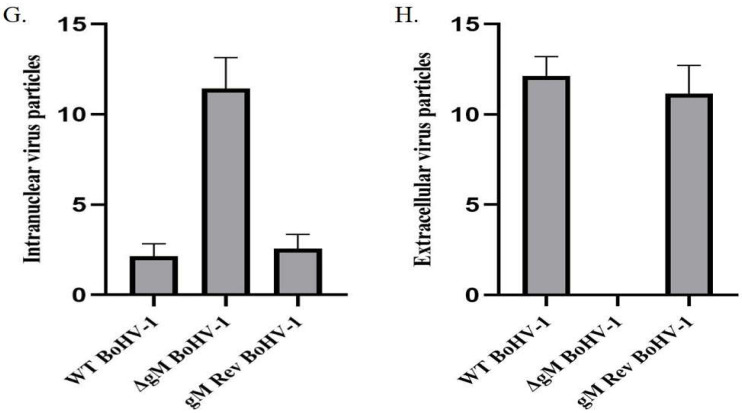
Transmission electron microscopy of cells infected with WT-BoHV-1, ΔgM BoHV-1, or ΔgM Rev BoHV-1, and processed at 7 h post infection. (**A**) A 20K magnified image of the nucleus of the cells infected with WT BoHV-1. A part of the nucleus is magnified 50K. The circles show the virus particles inside the nucleus. (**B**) A 6K magnified image of the cell membrane of the cells infected with WT BoHV-1. A part of the plasma membrane is magnified 20K. The circles show the virus particles outside the membrane and in between cells. (**C**) A 20K magnified image of the nucleus of the cells infected with ΔgM BoHV-1. A part of the nucleus is magnified 50K. The circles show the virus particles inside the nucleus. (**D**) A 6K magnified image of the cell membrane of the cells infected with ΔgM BoHV-1. A part of the cell membrane is magnified 20K. The circles show the virus particles egressing the nucleus; no virus was present on or outside the cell membrane. (**E**) A 20K magnified image of the nucleus of the cells infected with ΔgM Rev BoHV-1. A part of the nucleus is magnified 50K. The circles show the virus particles inside the nucleus. (**F**) A 6K magnified image of the cell membrane of the cells infected with ΔgM Rev BoHV-1. A part of the cell membrane is magnified 20K. The circles show the virus particles outside the cell membrane. The bars represent 0.2 µm. Mean values of the number of virus particles (**G**) inside the nucleus and (**H**) outside the cells in MDBK cells infected with WT BoHV-1, ΔgM BoHV-1, or ΔgM Rev BoHV-1, observed by TEM. Cells were infected at an MOI of 5 and fixed and processed 7 h post infection. The mean number of virus particles inside the nucleus and outside the cells ± SD of seven cells per sample is shown.

As the one-step growth kinetics of ΔgM BoHV-1, WT BoHV-1, and ΔgM Rev BoHV-1 appeared to show a delay in egress of the virus in ΔgM BoHV-1-infected cells, TEM was performed for confirmation. MDBK cells were infected with WT BoHV-1, ΔgM BoHV-1, or ΔgM Rev BoHV-1 at MOI 5 and examined at 7 h post infection (Figure 10A–F). The mean values of the number of virus particles observed inside the nucleus and outside the cells in cells infected with WT BoHV-1, ΔgM BoHV-1, or ΔgM Rev BoHV-1 are shown in Figure 10G,H, respectively. In the cells infected with WT BoHV-1, 2.14 ± 0.69 viral particles were observed inside the nucleus (Figure 10A,G) and 12.14 ± 1.71 at the plasma membrane, egressing the cells (Figure 10B,H). The observed ΔgM BoHV-1-infected cells showed 11.42 ± 0.79 virus particles inside the nucleus (Figure 10C,G), and none were found at the plasma membrane (Figure 10D,H). In ΔgM Rev BoHV-1-infected cells, the observed cells had 2.57 ± 1.07 virus particles inside the nucleus (Figure 10E,G) in contrast to 11.14 ± 1.57 virus particles outside the plasma membrane (Figure 10F,H), which is similar to a WT BoHV-1 infection. These observations showing more virus particles inside the nucleus than at the plasma membrane in ΔgM BoHV-1-infected cells when compared to the WT BoHV-1- or ΔgM Rev BoHV-1-infected cells further support the delay in egress and maturation of ΔgM BoHV-1 in infected cells.

### 3.6. VP8 Does Not Localize to the Golgi in the Absence of gM

**Figure 11:**
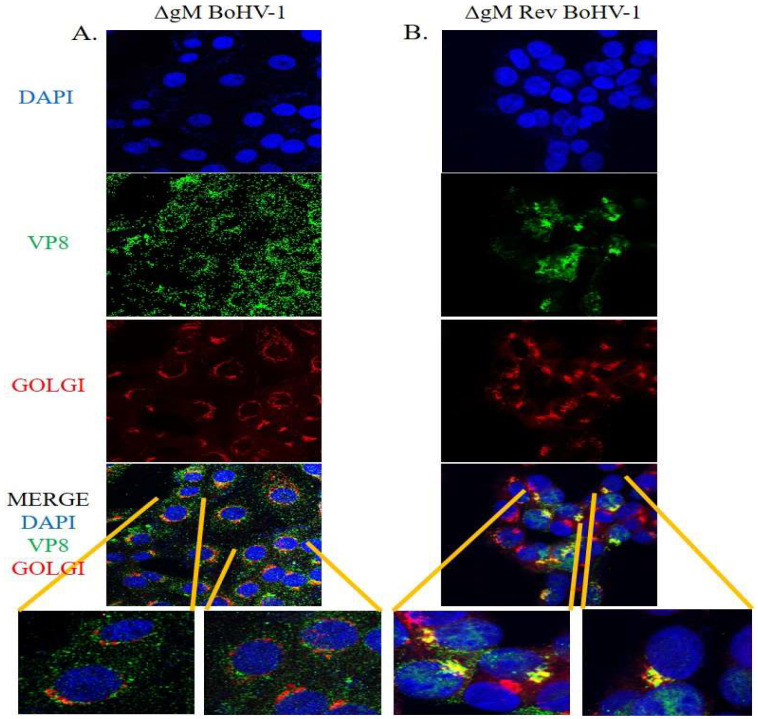
Localization of VP8 in cells infected with ΔgM BoHV-1 or ΔgM Rev BoHV-1. (**A**) Cells infected with ΔgM BoHV-1. VP8 is shown in green, and Golgi is shown in red. The merged image does not show any yellow which indicates that VP8 does not localize in the Golgi in the absence of gM. (**B**) Cells infected with ΔgM Rev BoHV-1. VP8 is shown in green, and Golgi is shown in red. The yellow in the merged image shows the localization of VP8 in the Golgi.

MDBK cells were infected with ΔgM BoHV-1 or ΔgM Rev BoHV-1 at a MOI of 5 to check the localization of VP8 to the Golgi at 7 h post infection. As shown in Figure 11A, VP8 was spread throughout the cytoplasm; accordingly, the merged image was devoid of any yellow, confirming that VP8 does not localize to the Golgi when MDBK cells are infected with ΔgM BoHV-1. However, when MDBK cells were infected with ΔgM Rev BoHV-1 (Figure 11B), the ability of VP8 to localize into the Golgi was restored, as shown by the yellow Golgi in the merged image. The restoration of the ability of VP8 to localize to the Golgi in the presence of gM confirms the indispensable nature of gM in the localization of VP8 to the Golgi, and possibly indirectly in the packaging of VP8.

### 3.7. The Amount of VP8 in Mature Virus Is Considerably Reduced in gM-Deleted Virus

**Figure 12:**
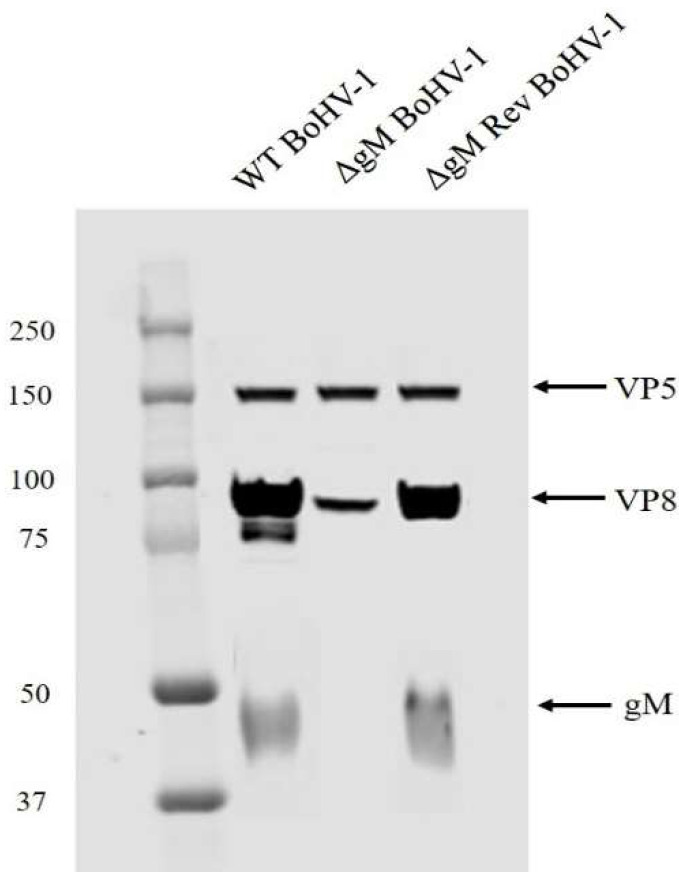
Amounts of VP8 present in virus purified from cells infected with WT BoHV-1, ΔgM BoHV-1, or ΔgM Rev BoHV-1. VP8 was detected using VP8-specific monoclonal antibody. The amount of VP8 was reduced to 33.8% in ΔgM BoHV-1 compared to 100% in WT BoHV-1 and 89.2% in ΔgM Rev BoHV-1.

To test the effect of gM on the final packaging of VP8, the amount of VP8 in the mature virus isolated from MDBK cells infected with ΔgM BoHV-1 was determined. MDBK cells were infected with WT BoHV-1, ΔgM BoHV-1, or ΔgM Rev BoHV-1 at a MOI of 5, and supernatants were collected at 18 h post infection. Virus was purified on a sucrose gradient as described previously [16], and the purified virus was subjected to Western blotting, with VP8-specific antibodies for detection (Figure 12). VP5 (capsid protein) was identified and used as a loading control on the gel. Based on densitometry analysis, the amount of VP8 in the mature ΔgM BoHV-1 was 33.8%, compared to 100% in the virus from cells infected with WT BoHV-1. In ΔgM Rev BoHV-1-infected MDBK cells, the amount of VP8 in the mature virus was found to be 89.2%. A considerable reduction in the amount of VP8 in the mature virus in the absence of gM and a major restoration in the amount of VP8 in ΔgM rev BoHV-1 confirms the indispensable role of gM in localization to the Golgi and hence packaging of VP8 into the virus.

## 4. Discussion

VP8 is the most abundant tegument protein of BoHV-1 and localizes to the nucleus early during infection. Following phosphorylation by US3 in the nucleus, it travels to the cytoplasm and finally localizes to the Golgi in BoHV-1-infected cells, where it is packaged into the mature virus [11]. However, VP8 does not localize to the Golgi outside the context of infection, indicating the involvement of a viral component in this process. The most predominant viral proteins found in the Golgi apparatus of the host cells infected with BoHV-1, and herpesviruses in general, are the viral glycoproteins. Viral glycoproteins play a variety of roles in the life cycle of a virus, ranging from virus entry into the host cells to virus assembly. In studies performed on HSV, it has been found that the incorporation of the tegument proteins into the virus depends on the interaction with other proteins, which are either capsid proteins, other tegument proteins, or glycoproteins [17]. Various tegument proteins interact with glycoproteins, which aid in their localization to the site of secondary envelopment, where the complete maturation of the virus takes place [18]. The site of secondary envelopment and virus maturation is the Golgi, where the majority of the glycoproteins/tegument proteins are packaged into the virions [19,20].

Based on these observations, the focus of this study was to identify the glycoprotein(s) involved in the subcellular localization of VP8 at the Golgi. VP8 was found to interact with gM in BoHV-1-infected MDBK cells (Figure 2). Glycoprotein M is encoded by different open reading frames in different herpes viruses. Glycoprotein M is encoded by the *U_L_10* gene in herpes simplex virus (HSV)-1 [21], HSV-2 [22], BoHV-1 [23], and pseudorabies virus [24]. In other alpha herpesviruses, gM is encoded by ORF50 (varicella zoster virus) [25] or ORF52 (equine herpes virus) [26]. Glycoprotein M exists as a complex with gN (encoded by *U_L_49.5*) in all herpesviruses via the formation of disulfide bonds [23,27]. The gM/gN complex has been shown to be critical in the packaging of several proteins during the maturation of BoHV-1 [28]. However, VP8 did not interact with gN. Glycoprotein M aids in the packaging of gN in HSV-1 [29] and pseudorabies virus [30]; however, in HSV gM, packaging is independent of gN [27]. In HSV, gM also aids in the packaging of several other proteins [31]; for example, tegument protein VP22, as VP22 packaging is impaired in the absence of gM [18]. Glycoprotein M promotes the cell-to-cell spread of several herpesviruses, including HSV-1 [29], BoHV-1 [32], varicella zoster virus [33], and equine herpesvirus-4 [34].

Immunoprecipitation studies with BoHV-1-infected (Figure 3) and VP8- and gM-cotransfected (Figure 4) cells were performed to further demonstrate an interaction between VP8 and gM; the results confirmed an interaction between these two proteins. Furthermore, VP8 and gM were shown to co-localize to the Golgi at 7 h post infection (Figure 5), which indicates a probable role of gM in the localization of VP8 to the Golgi. Previous studies demonstrated that in cells transfected with pFLAG-VP8 and US3 (viral kinase), VP8 moves from the nucleus to the cytoplasm but was not localized to the Golgi [11]. In the current study, the translocation of VP8 to the Golgi did not occur in cells transfected with pFLAG-ΔNLS VP8 alone (Figure 6A), which supports the inability of cytoplasmic VP8 to translocate to the Golgi without involvement of a viral factor. However, in EBTr cells cotransfected with pFLAG-ΔNLS VP8 and pHA-gM, both gM and VP8 localized to the Golgi (Figure 6C,D). The observation of gM enabling VP8 localization at the Golgi in the absence of any other viral factor is significant, suggesting that gM plays an important role in VP8 subcellular localization.

Domains are the functional units of a protein and play an important role in a protein’s function as well as interaction with another protein [15]. The domain between amino acids 482–632 in VP8 interacted with the domain between amino acids 210–300 in gM (Figure 7). In the secondary structures of these proteins obtained by psiPred software, these regions make up a helix–loop–helix structure, which is a signature structure of a functional domain (Figure 8). Furthermore, according to the predicted gM structure, amino acids 210–300 are located in the cytoplasm; this location agrees with the ability to interact with VP8. The deletion of the *U_L_10* gene from the virus significantly reduced the growth rate as well as the titer of both intracellular and extracellular virus (100-fold) (Figure 9), suggesting a delay in egress of ΔgM BoHV-1, which was confirmed by TEM studies performed on cells infected with WT BoHV-1, ΔgM BoHV-1, and ΔgM Rev BoHV-1 (Figure 10). In cells infected with ΔgM BoHV-1, the majority of the virus particles was found to be inside the nucleus, and none were found outside the cell. In contrast, in the cells infected with WT BoHV-1 and ΔgM Rev BoHV-1, the majority of the virus particles was outside the cells and very few were observed inside the nucleus (Figure 11). The reduction in the ΔgM BoHV-1 titer and the delay in egress from the ΔgM BoHV-1-infected cells could be correlated to the role of gM in the packaging of other tegument proteins like VP22 [28] or its importance in the cell-to-cell spread of BoHV-1 [32]. Furthermore, the localization of VP8 to the Golgi was hampered, and the amount of VP8 in the mature virus in cells infected with ΔgM BoHV-1 was reduced to 33.6%.This indicates that gM plays an important role in VP8 packaging (Figure 12), but that a portion of VP8 can be incorporated into virions during primary envelopment, which supports our previous results [8]. 

Based on this study, it can be concluded that gM plays an indispensable role in the subcellular localization and packaging of VP8. The growth rate and titer of ΔgM BoHV-1 are comparable to the growth rate of a BoHV-1 in which VP8 is not phosphorylated and does not exit the nucleus [8]. Given that VP8 is critical for viral replication, the slow growth rate and reduced titer of ΔgM BoHV-1 could be partially due to the reduced expression of VP8 and VP8′s inability to be translocated and hence packaged at the Golgi.

## Figures and Tables

**Table 1:** Primer list for construction of plasmids encoding HA-tagged truncated gM versions.

Plasmid	Forward Primer	Reverse
pHA-gM (70–438aa)	3′ATTTCTAGAATGGGCGCGCGCCACCCGGCGCT5′	3′ATGATATCTTTGACGTGCGCGGGGGGTGGG5′
pHA-gM (140–438aa)	3′ATTTCTAGAATGACCGCCGGGCTGCCCGGCGC5′	3′ATGATATCTTTGACGTGCGCGGGGGGTGGG5′
pHA-gM (210–438aa)	3′ATTTCTAGAATGCTGGGGCTGTCGCTGGCACA5′	3′ATGATATCTTTGACGTGCGCGGGGGGTGGG5′
pHA-gM (300–438aa)	3′ATTTCTAGAATGGCCCCCCGGGCTGCCGCTAG5′	3′ATGATATCTTTGACGTGCGCGGGGGGTGGG5′
pHA-gM (250–438aa)	3′ATTTCTAGATGGTCGCCGGCGTGACGG5′	3′ATGATATCTTTGACGTGCGCGGGGGGTGGG5′
pHA-gM (140–264aa)	3′ATTTCTAGATGGCGCTGGCGGCCT5′	3′ATGATATCTTTCAAAAAGAGCACGGCGG5′

**Table 2:** Primer list for construction of ΔgM BoHV-1 and ΔgM Rev BoHV-1 recombinant viruses.

Primer Name	Primer Sequence
Primer 1	3′GGAGGTACCGTATTACCGCCATGCATTAG5′
Primer 2	3′GAGGGATCCTGCCGATTTCGGCCTATTGG5′
Primer 3	3′GCGGAATTCGCGCTGCATCTCGTCACTTTCATCG5′
Primer 4	3′GAAGGTACCCGCCAACCATACCGCTAAGGAGACC5′
Primer 5	3′GAGAAGCTTCGTAAAGCTGCGCCGACAGGAG5′
Primer 6	3′CTGCGCGGAGCCCGCGATGACGGCAAC5′
Primer 7	3′GAAGGATCCCGCCAACCATACCGCTAAGGAGACC5′
Primer 8	3′GGCGTTGCCGTCATCGCGGGCTCCGCGCAG5′

## Data Availability

Not applicable.
